# Climate-Induced Range Shifts and Possible Hybridisation Consequences in Insects

**DOI:** 10.1371/journal.pone.0080531

**Published:** 2013-11-15

**Authors:** Rosa Ana Sánchez-Guillén, Jesús Muñoz, Gerardo Rodríguez-Tapia, T. Patricia Feria Arroyo, Alex Córdoba-Aguilar

**Affiliations:** 1 Departamento de Ecología Evolutiva, Instituto de Ecología, Universidad Nacional Autónoma de México, México D.F., Mexico; 2 Real Jardín Botánico (RJB-CSIC), Madrid, Spain; 3 Unidad de Geomática, Instituto de Ecología, Universidad Nacional Autónoma de México, México D.F., Mexico; 4 Department of Biology, The University of Texas-Pan American, Edinburg, Texas, United States of America; 5 Centro de Biodiversidad y Cambio Climático, Universidad Tecnológica Indoamérica, Quito, Ecuador; CNRS, France

## Abstract

Many ectotherms have altered their geographic ranges in response to rising global temperatures. Current range shifts will likely increase the sympatry and hybridisation between recently diverged species. Here we predict future sympatric distributions and risk of hybridisation in seven Mediterranean ischnurid damselfly species (*I. elegans*, *I. fountaineae*, *I. genei*, *I. graellsii*, *I. pumilio*, *I. saharensis* and *I. senegalensis*). We used a maximum entropy modelling technique to predict future potential distribution under four different Global Circulation Models and a realistic emissions scenario of climate change. We carried out a comprehensive data compilation of reproductive isolation (habitat, temporal, sexual, mechanical and gametic) between the seven studied species. Combining the potential distribution and data of reproductive isolation at different instances (habitat, temporal, sexual, mechanical and gametic), we infer the risk of hybridisation in these insects. Our findings showed that all but *I. graellsii* will decrease in distributional extent and all species except *I. senegalensis* are predicted to have northern range shifts. Models of potential distribution predicted an increase of the likely overlapping ranges for 12 species combinations, out of a total of 42 combinations, 10 of which currently overlap. Moreover, the lack of complete reproductive isolation and the patterns of hybridisation detected between closely related ischnurids, could lead to local extinctions of native species if the hybrids or the introgressed colonising species become more successful.

## Introduction

Numerous studies addressing species’ responses to climate change, mostly in animals, have provided evidence of altered geographic ranges in response to rising global temperatures [1,2]. In Europe, for example, many cases of range shifts among insects have been attributed directly to increasing temperatures [1]. One consequence of range shifts is an increased sympatry between recently diverged species, likely increasing the potential for interspecific interactions and hence hybridisation [3]. 

Several contemporary examples of hybridisation between native and introduced species after range expansion include insects such as the brown argus butterflies in Britain [4], mammals such as grizzly and polar bears (*Ursus arctos* and *U. maritimus*) [5], flying squirrels (*Glaucomys sabrinus* and *G. volans*) [6], and fishes such as westslope cutthroat trouts (*Oncorhyncus clarki lewisi* and *O. mykiss*) [7]. Interspecific matings can actually initiate a first generation of hybrids (F_1_) if reproductive isolation barriers are not complete [1]. Furthermore, if F_1_ hybrids backcross with at least one of the parental genotypes and the resulting backcrossed individuals subsequently mate with the most similar parental genotype, novel genes can be rapidly introduced into the new genetic background [8]. The consequence of this situation is a massive introgression of genes between local and invading species [9], forming stable and long-lasting hybrid zones [10-12]. Several of the adaptive processes through hybridisation are reinforcement, adaptation and speciation [13,14]. However, another possible consequence is the local extinction of native species which occur when the new hybrid or the invading species is more successful and thus displaces the native species [4]. 

Odonates (dragonflies and damselflies) are good dispersers that frequently leave their native habitat after emergence to colonize new ponds and/or rivers [15,16]. Members of this order are undergoing a northward range expansion, apparently in response to climatic changes [17,18]. In Great Britain all but three of the 41 non-migratory species have shifted their distribution northward at their range margin by 12–346 km [17]. In odonates, premating reproductive barriers seem to evolve independent of niche diversification [19-21], i.e. speciation can occur without niche diversification. However, studies in ischnurid damselflies (Coenagrionidae) have revealed extensive hybridisation between sister species and local extinction for the native species [e.g. 22,23].

In this paper, we have explored the risk of extirpation of native species by the invasion of close relatives in response to climate change in seven Mediterranean ischnurid, odonate species (*I. elegans*, *I. fountaineae*, *I. genei*, *I. graellsii*, *I. pumilio*, *I. saharensis* and *I. senegalensis*). Among odonates, Mediterranean ischnurids are a good model to understand how insects can cope with on-going climate and environmental change because: 1) the Mediterranean region is expected to experience a major loss of biodiversity due to the joint action of invasive species, habitat fragmentation and climate change [24], and 2) many aspects of ischnurid ecology have been investigated in detail such as range expansions [17], ecological factors shaping their distributions [25] [for environmental and climatic determinants for the distribution in Ischnura *elegans* see 25], and reproductive isolation [23,26-29]. 

We carried out our study in two steps. First, we constructed distribution model projections for the present time and the years 2020, 2050 and 2080 following the IPCC climate predictions [30], to detect expanding, contracting and new overlapping ranges of the seven Mediterranean ischnurids. Second, we did a comprehensive data compilation of reproductive isolation (habitat, temporal, sexual, mechanical and gametic) between the seven studied species. Merging these two information pieces allowed us to infer the risk of hybridisation in these animals. This methodology illustrates one way to predict hybridisation risk related to the rise of global temperatures that could be applied to other taxonomic groups.

## Materials and Methods

### Study area and environmental predictors

**Figure 1:**
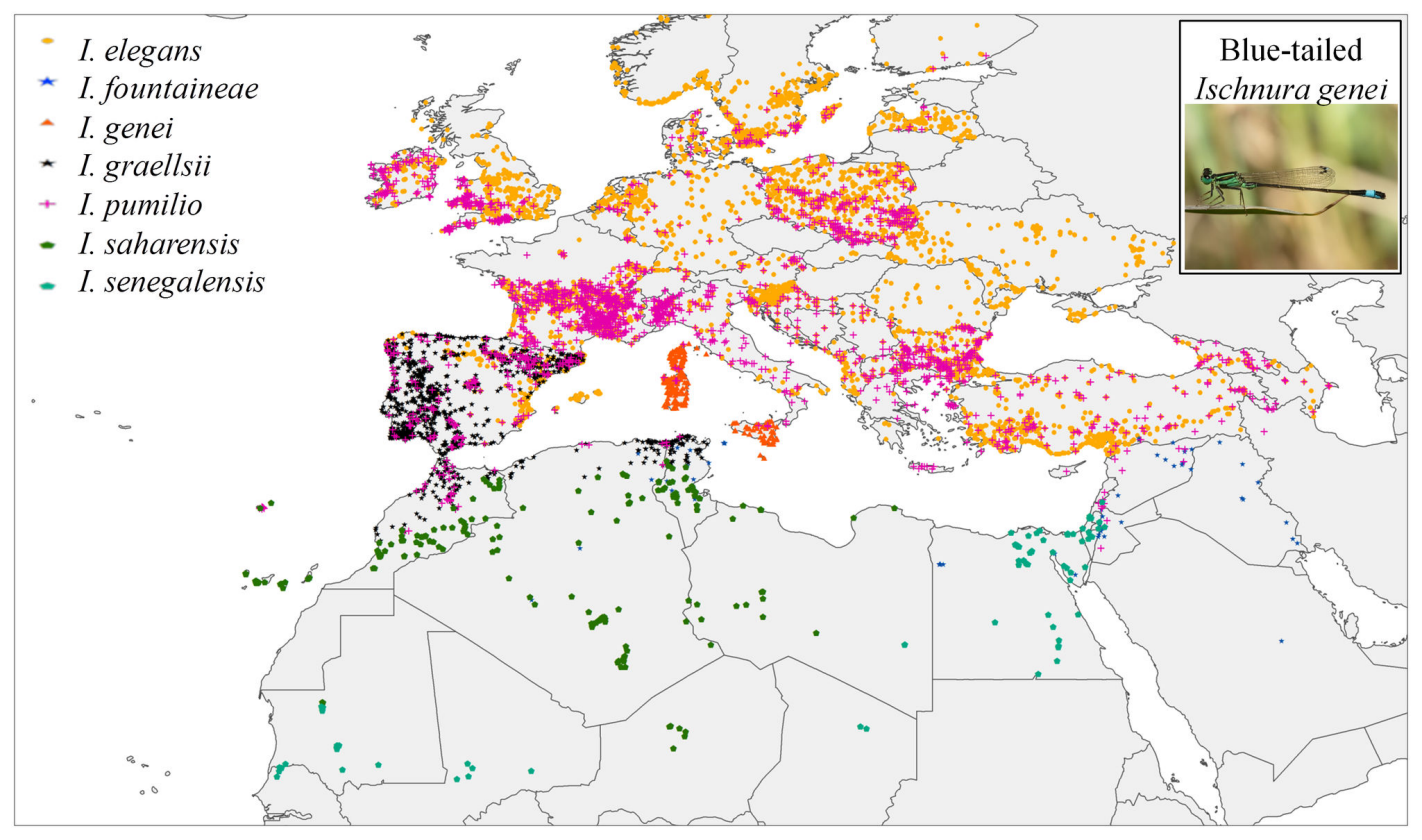
Distribution map of the seven Mediterranean *Ischnura* species treated in this study (*I. elegans*, *I. fountaineae, I. genei*, *I. graellsii, I. pumilio, I. saharensis* and *I. senegalensis*).

The spatial framework included all European (west of Russia) and North African countries where members of the genus *Ischnura* occur (Figure 1). As bioclimatic variables we used the WorldClim 1.4 (www.worldclim.org) data set [31] at 5 × 5 km cell size. We visually inspected the variables and eliminated those with strange patterns in the study area, mostly due to the difficulty of getting reliable interpolated precipitation values across the Sahara desert (bio_2, bio_3, bio_8, bio_9, bio_15, bio_18 and bio_19). To establish a set of uncorrelated climatic variables, we intersected the remaining variables with 10,000 points randomly selected in the extension of the study area, ran an exploratory data analysis and a correlation analysis, and eliminated one of the variables in each pair with a Pearson correlation value > 0.7. The final data set includes only Annual Mean Temperature (bio_1), Temperature Annual Range (bio_7) and Annual Precipitation (bio_12). We generated a second set of models with the first 2-6 components as predictors after running a Principal Component Analysis, and a third set using all WorldClim variables.

### Species distributional data

Presences from the strictly Mediterranean countries consisted of 11,376 records [32-44]. This data set is biased with regard to *I. elegans* and *I. pumilio* in two ways: it lacks presences from their northern and eastern European distributions, and it has a large number of presences concentrated in areas that have been more thoroughly sampled studied. To correct for these biases, we completed distribution gaps with presences downloaded from GBIF (www.gbif.org; accessed 3-Mar-2013) and data gathered from several country-level data sets or publications without records in GBIF: Armenia (Vasil Ananian and Marc Tailly, and www.armenodon.org); Azerbaijan and Georgia (Marc Tailly); Finland (Sami Karjalainen), Latvia (Kalninš M. and [45]); Lithuania (Rafał Bernard); Poland (Rafał Bernard and [46]); Romania (Cosmin O. manci); Czech Republic [47], Slovenia (Ali Salamun), Slovakia (Dušan Šácha), and Ukraine (Elena Dyatlova). After merging all presences together, we perform a subsample of *I. elegans* presences to reduce the geographical sampling bias. The final data set thus included seven species (Figure 1): *I. elegans* (4089 unique presences at 5 × 5 km pixel size), *I. fountaineae* (109), *I. genei* (184), *I. graellsii* (1081), *I. pumilio* (2139), *I. saharensis* (178) and *I. senegalensis* (78). In most of the Mediterranean area, *I. elegans* and *I. pumilio* occur in sympatry. Species of the *I. elegans* group (*I. elegans*, *I. genei*, *I. graellsii* and *I. saharensis*) and *I. fountaineae* overlap locally: *I. elegans* and *I. graellsii* in Spain; *I. elegans* and *I. genei* in Elba and Giglio Islands; and *I. graellsii* and *I. saharensis* in Morocco [36,48]. Additionally, *I. fountaineae*, *I. saharensis* and *I. graellsii* overlap in Morocco, while *I. elegans, I. fountaineae, I. pumilio* and *I. senegalensis* overlap in the Middle East [36,48]. Table 1 shows range, habitat, flight season and IUCN Red List Status for each species.

**Table 1:** Range, habitat, flight season and IUCN (2012.2) Red List Status for the species used in this study.

**Species**	**Range**	**Habitat**	**Flight season**			**References**	
***I. elegans***	From Ireland to the Mediterranean and to Japan	Running and standing waters Tolerant to brackish waters	(March) April-October (November). 1-3 generations/year			[36,85]	
***I. fountaineae***	From SW Asia to the Middle East and the West of Maghreb	Running and standing waters Tolerant to high salinity	March-November Several generations/year			[36,85]	
***I. genei***	Tyrrhenian endemic: Corsica, Sardinia, Sicily, Tuscan archipelago, Malta	Running and standing waters	April-October			[36,37,85]	
***I. graellsii***	Western Mediterranean area: Iberia and Maghreb	Running and standing waters Tolerant to brackish waters	March-November. 1-4 generations/year			[36,85]	
***I. pumilio***	From the Azores to W Mongolia and the North of the Maghreb	Running and standing waters	April-October. 1-3 generations/year			[36,85]	
***I. saharensis***	Sahara from Mauritania and Niger to North East Libya and the Maghreb; Canary Islands	Running and standing waters Tolerant to brackish waters	February-December. At least 2 generations/year			[36,85]	
***I. senegalensis***	From Africa to Japan and western New Guinea. One valid record from Tenerife	Running and standing waters Tolerant to brackish waters	All year round where possible			[36]IUCN	

Based on the assumption that odonate distribution is mainly affected by ecophysiological traits [49], we did not use topographical variables to determine species topographic limits. For instance, distributional range limits for the odonates *Calopteryx splendens* and *C. virgo* are mainly driven by physiological temperature and precipitation optima [21]. This led Wellenreuther et al. [21] to propose that future climate change will largely affect odonate distribution ranges. On the other hand, bioclimatic (mean annual temperature and precipitation) but not geographic variables (altitude and distance to coast) have been detected to explain genetic population structure in the damselfly *Ischnura elegans* [25], and to explain current and future distributions in *Schistolobos boliviensis* and *Tuberculobasis inversa* [50]. Mediterranean ischnurids are ecologically similar, and species share similar habitat types characterized by running and standing waters and, except for *I. pumilio* and *I. genei*, all are tolerant to brackish waters (ranges, habitat and flight seasons are detailed in Table 1).

### Construction of species distribution models

Species distribution models were generated with Maxent 3.3.3k [51], a deterministic algorithm that has been shown to be among the best modelling methods [52,53]. Models were constructed setting several parameters to default (‘Auto features’, convergence = 10^-5^, maximum number of iterations = 500) and varying the prevalence (0.5, 0.6 and 0.7) and regularization value β (1, 2 and 3) to find which combination generated the best outcomes (highest Area Under the curve, or AUC) while minimizing the number of model parameters, as well as producing ‘closed’, bell-shaped response curves guaranteeing model transferability. A regularization multiplier higher than 1.0 allows that variables’ average values in the projections spread from the empirical average of the background points (the situation if it is set to 1.0), avoid model overfitting [54], and smooth the response curves. Regarding background, we experimented with several selection schemes: 1) randomly selecting 10,000 point from areas adjacent to presences and pertaining to the same Köppen-Geiger bioclimatic region and elevation range as the species being modelled [55-57]; 2) randomly selecting 10,000 points; or 3) randomly selecting 40,000 points in the whole of the study area (Figure 1). In total, 81 models were generated for each species (3 variables schemes × 3 regularization × 3 prevalence × 3 background schemes). Performance of the models was assessed by means of the AUC in a ROC statistic through 10-fold cross-validation (Table 2), and minimizing the number of model parameters.

**Table 2:** Performance of the models, measured as the average testing AUC of 10-crossvalidation replicates, and change in range area.

**Species**	**AUC**	**A2a-2020**	**A2a-2050**	**A2a-2080**
*I. elegans*	0.9115	-11.85	-26.01	-33.44
*I. fountaneae*	0.9285	-25.38	-40.81	-45.13
*I. genei*	0.9951	-70.01	-77.86	-77.13
*I. graellsii*	0.9643	23.53	34.35	57.20
*I. pumilio*	0.9218	-6.77	-23.20	-23.17
*I. saharensis*	0.9081	-28.16	-45.06	-57.44
*I. senegalensis*	0.9091	-46.78	-70.18	-83.29

Percentage of range loss and range gain with respect to current potential range for each future projection (2020, 2050 and 2080) under scenario A2a; each year is represented by a consensus model where only pixels predicted present by the four GCMs are considered as presence of the species.

Continuous outcomes (Maxent models) were transformed to presence/absence models using the ‘10 percentile training presence’. Although recent research has shown that ‘maximum training sensitivity plus specificity’ is a preferable threshold selection method in presence-only models [58], the origin of our data set caused georeferencing issues in a number of presences, and we prefer to err in the side of caution accepting that a 10% of our presences could be problematic.

The best models for current climatic conditions were used to generate high resolution maps of likely range shifts due to climate change. We used the A2a scenario to generate future projections, as this scenario seems to be the more realistic at present [59]. As there are a number of Global Circulation Models available, and no one can be considered superior to the rest, we generate future projections for the four GCM with data available for the time slices 2020, 2050 and 2080 at the CGIAR Research Program on Climate Change, Agriculture and Food Security (CCAFS) spatially downscaled using the Delta Method (http://www.ccafs-climate.org): CCCMA-CGCM2, CSIRO-MK2.0, UKMO-HADCM3, and NIESS99). The final presence/absence model for each species and time slice was the area where the four models predicted presence for the species considered (Figure 1). To confirm that combinations of novel climates were not cause of concern in the projections, we generated the multivariate environmental similarity surfaces (MESS); this grid was reclassified and values below zero were masked to show areas of novel climate space relative to the range under which the model was fitted (Figures S1-S7).

We simulated predicted current and future ranges, i.e. we simulated the areas with current and future climate conditions equivalent to those of its present range. This does not mean that the species will occupy all these areas as we did not take into account their population dynamics, dispersal abilities or habitat availability [see 60 for similar trends]. Thus, in order to reduce any effect of model bias, predicted future distribution extent and predicted overlapping ranges among the likely hybridizing species were calculated relative to the species’ range simulated in the same way for the current climate for the seven studied species. Expansion or contraction of the distribution ranges for the three time slices were estimated as the number of predicted km^2^ that each species will occupy for each one divided by the number of km^2^ that each species will occupy at the current predicted binary distribution. Increases or decreases of the overlapping ranges were estimated at a similar form: the number of predicted km^2^ that each pair of species will overlap for each time slice divided by the number of predicted km^2^ that each pair of species will overlap at the current predicted binary distribution.

### Species’ relevant biology and data collection of reproductive isolation

Ischnurines can share the same geographic area but still be isolated by a fine-scale, subtle habitat-species association [23]. Moreover, species can also be isolated in time due to species-specific phenology and daily mating activity. When species overlap in space and time, they can become isolated via sexual isolation, which refers to mate preferences toward conspecifics [61,62]. Furthermore, in odonates, prior to mating, the male must grasp the female by her prothorax (tandem) using his anal appendages [63]. After that, a female must bend her abdomen to allow genital contact (mating). The incompatibility to achieve the tandem and the mating position is named mechanical incompatibility [64,65]. Once mating has finished, females will lay eggs if the insemination, gametes’ recognition and fertilization are successful, i.e. if there is not gametic isolation. In this study, we did a comprehensive data compilation of reproductive isolation between the seven studied species. Data was grouped on the above categories of isolation: habitat, temporal, sexual, mechanical and gametic based on a previous study, on which reproductive barriers (19 pre- and postmating barriers) between two ischnurids (*I. elegans* and *I. graellsii*) were measured and categorised [see 23]. In addition, when two species have not been in contact before, or there is not information about reproductive isolation, their hybridisation potential was predicted based on the positive correlation between genetic divergence and reproductive isolation. This correlation was previously detected in damselflies [66] and other organisms [67-71]. We predict the risk of hybridisation in those species (susceptible to hybridise) potentially experiencing sympatry according to our future climatic predictions. Data for each species according to range, habitat, and flight season have been compiled in Table 1. 

## Results

### Predicted current and future distribution of the studied species

For each species, we selected for further analyses the models combining high performance (high AUC) and transferability (i.e. low number of parameters and “closed” curves). For all species, optimal models were those trained with the uncorrelated original variables and 10,000 background points. Optimal models for *I. elegans*, *I. genei*, *I. pumilio* and *I. senegalensis* were generated using the three variables (Annual Mean Temperature, Temperature Annual Range and Annual Precipitation), while Temperature Annual Range was not used for *I. fountaineae*, *I. graellsii* and *I. saharensis*. Using more background points either diminished model performance or increase model complexity, and using PCA components as variables strongly increased models’ complexity, compromising transferability. 

**Figure 2:**
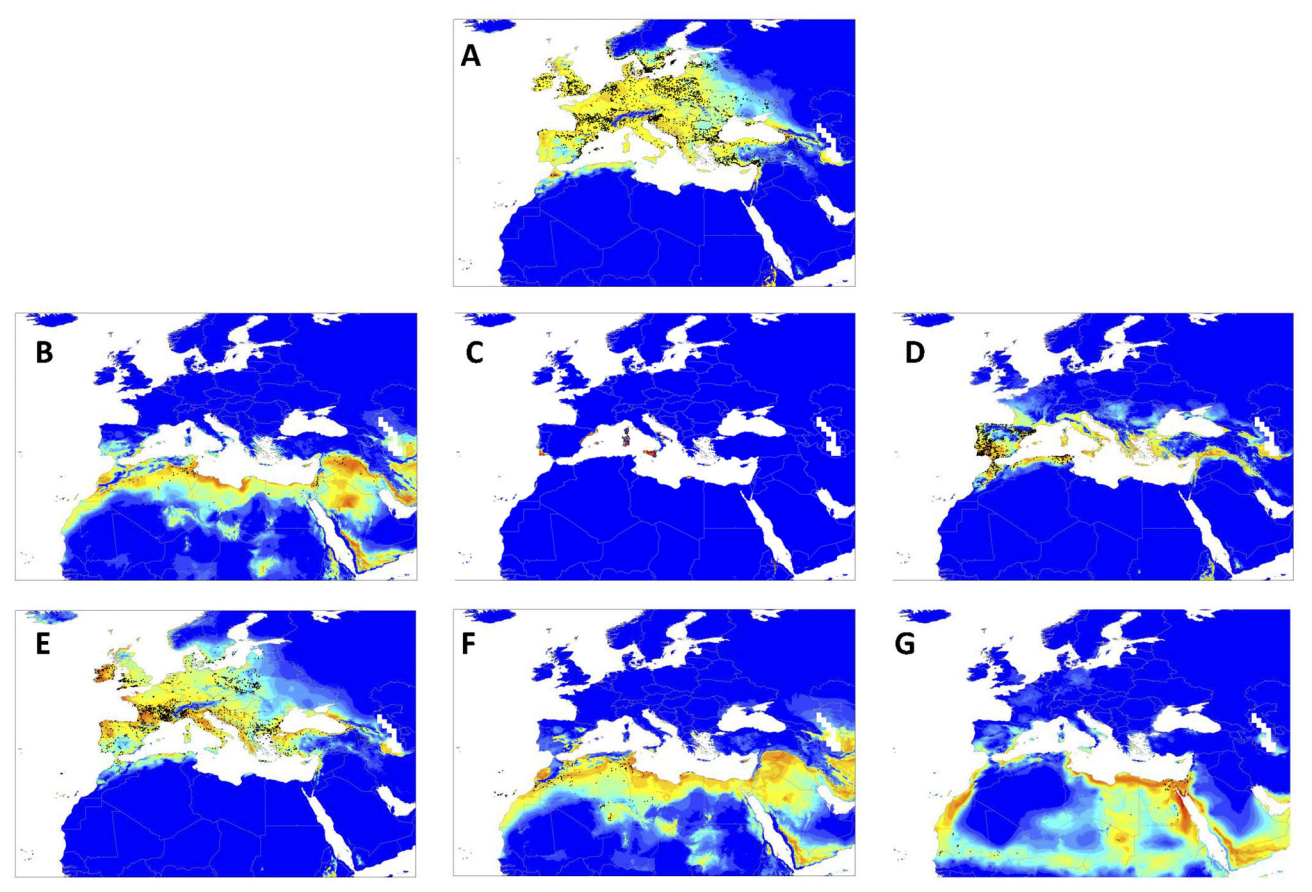
Suitability map for current climatic conditions and unique presences used to generate the models of the seven Mediterranean *Ischnura* species treated in this study: *I. elegans* (A); *I. fountaineae* (B); *I. genei* (C); *I. graellsii* (D); *I. pumilio* (E); *I. saharensis* (F); *I. senegalensis* (G). Suitability increases from dark blue (0) to green (0.5) to red (1).

Optimal model performance based on the AUC had a mean score of 93.4%, ranging from 0.900 ± 0.024 for *I. elegans* to 0.995 ± 0.002 in *I. graellsii*. Predicted current distribution modelling for the seven species indicated extensive areas of suitable habitat in the Mediterranean region which are not currently occupied (Figure 2). 

Distribution models projected to scenario A2a for the three periods 2020, 2050 and 2080 (Figures S1-S7) indicated an extensive decrease of suitable habitat for all species (from 6.77 to 83.29%), except for *I. graellsii* (Table 2), which is predicted to increase its potential distribution (from 23.53 to 57.20%). The future distribution models for the three periods were consistent, showing a progressive decrease or increase of the suitable habitats for all species. 

Moreover, future distributional models indicated a shift of the suitable areas toward northern latitudes for all species except for *I. senegalensis* (see Figures S1-S7). Pairwise overlapping distribution ranges were estimated for the seven species. Future distributional models predicted an increase of the potential overlapping ranges in 12 out of the 42 species combinations (Table 3). *Ischnura graellsii* is the only species that will increase its potential distribution, and is thus the species which will increase its overlapping range with most other species: *I. elegans*, *I. fountaineae*, *I. genei*, *I. pumilio* and *I. saharensis. I. genei* is the species that will be overlapped by the most others (3 species), although *I. elegans* (2), *I. fountaineae* (2), *I. saharensis* (2) *I. senegalensis* (2) and *I. pumilio* (1) will also be overlapped (Table 3).

**Table 3:** Change in overlapping areas.

**Overlapped species**	**Overlapping species**	**Potential actual**	**Potential 2020**	**Potential 2050**	**Potential 2080**
*I. elegans*	*I. fountaineae*	5.28	4.99	1.95	0.09
	*I. genei*	4.12	1.41	1.34	1.57
	***I. graellsii***	28.09	**28.98**	**28.08**	**33.87**
	*I. saharensis*	1.44	0.49	0.04	0.00
	***I. pumilio***	86.24	**87.23**	**88.65**	**90.78**
	*I. senegalensis*	3.30	2.27	0.49	0.08
*I. fountaineae*	***I. elegans***	5.72	**6.38**	2.64	0.12
	*I. genei*	1.38	0.54	0.03	0.00
	***I. graellsii***	11.68	**15.94**	10.82	2.99
	*I. saharensis*	92.68	86.45	78.25	74.83
	*I. pumilio*	5.16	3.54	0.88	0.04
	*I. senegalensis*	35.48	27.78	20.73	9.76
*I. genei*	***I. elegans***	89.23	**89.87**	**96.92**	**99.34**
	*I. fountaineae*	27.66	27.12	1.60	0.03
	***I. graellsii***	93.10	**94.43**	92.68	**97.53**
	*I. saharensis*	9.39	4.57	0.15	0.06
	***I. pumilio***	86.68	82.28	**97.94**	**99.82**
	*I. senegalensis*	35.21	32.10	2.00	0.00
*I. graellsii*	*I. elegans*	71.55	52.69	39.39	36.53
	*I. fountaineae*	27.49	22.66	11.21	2.45
	*I. genei*	10.94	2.69	1.79	1.67
	*I. saharensis*	16.28	10.11	5.05	1.35
	*I. pumilio*	74.75	60.40	40.48	40.62
	*I. senegalensis*	12.45	5.26	1.08	0.08
*I. saharensis*	*I. elegans*	1.28	0.54	0.04	0.00
	***I. fountaineae***	76.48	74.10	69.56	**79.61**
	*I. genei*	0.39	0.08	0.00	0.00
	***I. graellsii***	5.71	**6.10**	4.33	1.74
	*I. pumilio*	1.67	0.20	0.03	0.02
	*I. senegalensis*	33.79	26.98	20.33	7.30
*I. pumilio*	*I. elegans*	95.11	90.96	94.18	86.74
	*I. fountaineae*	5.25	2.88	0.69	0.03
	*I. genei*	4.41	1.35	1.44	1.51
	***I. graellsii***	32.36	**34.65**	30.66	**35.99**
	*I. saharensis*	2.06	0.19	0.03	0.01
	*I. senegalensis*	3.45	1.41	0.18	0.01
*I. senegalensis*	*I. elegans*	3.19	3.64	1.17	0.30
	***I. fountaineae***	31.75	**34.86**	**36.82**	28.69
	*I. genei*	1.57	0.81	0.07	0.00
	*I. graellsii*	4.74	4.65	1.85	0.29
	***I. saharensis***	36.65	**39.49**	**40.63**	20.17
	*I. pumilio*	3.03	2.17	0.42	0.03

Percentage of overlapping areas between each future projection (2020, 2050 and 2080) under scenario A2a and the actual potential occupied range. The values highlighted in bold indicate where the overlapping of predicted future distributions increase relative to overlapping of predicted current distributions.

### Reproductive isolation between the studied species

We detected interspecific interactions between 10 species combinations comprising five out of the seven studied, with hybrids being detected in seven species combinations [23,26,27,66,72-75] (see Table 4). Risk of hybridisation between the remaining species combinations and cross directions (31) was based on genetic distances. Pairwise genetic distances between *Ischnura elegans, I. fountaineae, I. genei*, *I. graellsii* and *I. saharensis* fall within the threshold for hybridisation, and thus are susceptible to hybridise if they come into contact [see 66 for further details]. In other words, although the number of species is relatively low, our review implies that hybrid production may be fairly common in these animals. 

**Table 4:** Summary of heterospecific interactions, and the susceptibility to hybridise.

**Species**	***♀ elegans***	***♀ genei***	***♀ graellsii***	***♀ saharensis***	***♀ fountaineae***	***♀ pumilio***	***♀ senegalensis***
***♂ elegans***		H_I_, H_II_	H_I_, H_II_	Yes	yes	AT, T and M	No
***♂ genei***	AT, T and M		H_I_,	Yes	Yes	Yes	No
***♂ graellsii***	H_I_, H_II_	Yes		H_I_, H_II_	Yes	Yes	No
***♂ saharensis***	Yes	yes	H_I_, H_II_		yes	No	No
***♂ fountaineae***	Yes	Yes	Yes	Yes		No	No
***♂ pumilio***	AT	No	AT, T and M	No	No		No
***♂ senegalensis***	No	No	No	No	No	No	

Attempt to tandem (AT) (male attempt to grasp the female but tandem (T) is impeded due to incompatibility of both sexes’ secondary genitalia), mating (M) (mating takes place but female does not oviposit), hybrids I (H_I_) (i.e. hybrids obtained under laboratory conditions) and hybrids II (H_II_) (hybrids genetically detected in field conditions) [23,26-28,73-75]. When no data about reproductive isolation was found, mainly in allopatric species combinations, we approximate the risk of hybridisation based on genetic distances [see 66]: “Yes” means that species may produce hybrids, and “No” means that species cannot produce hybrids if they come into contact.’.

## Discussion

Species distribution models are used to address a wide variety of ecological and evolutionary issues [76]. Here we explore the role that global change could play in the extirpation of native species by the invasion of close relatives, either because hybrids or range overlapping. Our projections, under different Global Circulation Models, predicted a general northward and westward displacement, and a decrease in potential distribution ranges for all species except *I. graellsii*. According to these results, range shifts will give rise not only to new overlapping areas for current sympatric species, but also create new sympatric distributions for species that have evolved in allopatry [3,4,26]. Our results predicted that 12 out of the 42 *Ischnura* species combinations that overlap in their actual potential occupied ranges would increase their overlapping range at least in one direction, although only 10 pairs currently overlap. 

Our predictions are in agreement with previous studies which have detected a shift of range borders of several African and Mediterranean odonate species which are extending northwards to central and northern Europe [18], and also westward under the influence of climate change. For instance, *I. elegans* and *I. pumilio* are undergoing a northward range expansion in the United Kingdom [17], and *I. elegans* has spread to the west regions of Spain [26-28]. However, not all studied species will undergo range expansions: *I. senegalensis* will gradually lose most of its current suitable area in inland Africa without a parallel northward expansion in Europe. This is because the extremely hot conditions where it currently lives will not be reached in Europe before 2080. Similarly to what is expected for *I. elegans* and *I. pumilio*, a poleward range shift of future distributions caused by rising temperatures has been predicted for several Mediterranean species (e.g. bats), and some possible consequences are extinction and decline patterns under A1F1 emission scenario [77]. Whether this will be the case for our study species, is unclear. However, notice that our study assumes eco-physiological parameters but not biotic factors to drive ranges, and thus, likely future distributions. Distributions may be affected in at least some of these species, implying that competition would limit invasion by one species thereby reducing or eliminating the potential for hybridisation. 

Range shifts can lead to the formation of or increase in sympatry between related organisms, which consequently will increase the potential for sexual interactions and hybridisation. Five out of the 10 pairs of species predicted to increase in their potential overlapping ranges, are also predicted to hybridise: *I. elegans* and *I. genei; I. elegans* and *I. graellsii*; *I. graellsii* and *I. saharensis*; *I. fountaineae* and *I. saharensis*; and *I. fountaineae* and *I. graellsii*). Several factors such as a) ecological partitioning [78], b) time of divergence [67-71], c) specific stage of reproductive isolation [23,26], and d) species abundance [79] shape hybridisation patterns. We discuss each of these factors below. 

Ecological partitioning among species can prevent competitive exclusion [78]. However, niche space reduction is especially true for odonates; in fact, speciation in these animals takes place without niche divergence [21,80,81]. Two examples can illustrate these. First, all of our study species share their habitat preference for running and standing waters, and only *I. elegans*, *I. graellsii* and *I. saharensis* are able to tolerate brackish waters. Second, all share an extended reproductive season from April to October [36]. This lack of microhabitat and temporal isolation can thus lead to many opportunities for interspecific interactions (see Table 3) and the creation of stable and long-lasting hybrid zones when reproductive isolation breaks [12], as documented for *I. elegans* and *I. graellsii* in the Iberian Peninsula [26], *I. graellsii* and *I. saharensis* in North Africa, and *I. elegans* and *I. genei* in Tyrrhenian islands [66]). In relation to divergence time and specific stage of reproductive isolation, genetic divergence correlates positively with reproductive isolation between recently speciated species, and this correlation can be used to predict hybridisation [67-71]. In damselflies, a positive correlation between genetic divergence and reproductive isolation has been detected [66]. In fact, the pairwise genetic distances between five species (*I. elegans, I. fountaineae, I. genei*, *I. graellsii* and *I. saharensis*) fall within the threshold for hybridisation [66]. Finally, in relation to species abundance, females will rarely mate with heterospecific males if these males are less abundant than conspecific males, based on a) the higher inversion on offspring production by females compared to males [82] and b) mechanical incompatibility that arises from smaller heterospecific males compared to conspecific males [83]. Nevertheless, females will mate with heterospecific males, when these are the most abundant males in the population, so as to ensure fertilization. However, this was not the case for *I. elegans* when invaded *I. graellsii* populations [26]. In this case, *I. graellsii* females mated with heterospecific males *I. elegans* which turned out to be less abundant that conspecific *I. graellsii* males [26].When hybrid formation is not prevented by previous barriers, and hybrids mate among each other and they become more successful than one or both parental species, original taxa can be displaced [14]. However, in the majority of the cases one of the two species is displaced by others via unidirectional hybridisation, i.e. backcrossed individuals consecutively mate with the more similar species [84]. Although this panorama seems speculative, it explains well why Spanish populations of *I. graellsii* are currently being displaced by *I. elegans* via unidirectional introgression of genes of *I. graellsii* into *I. elegans* [23]. However, it is unknown whether the introgression of genes of *I. graellsii* in *I. elegans* is helping *I. elegans* to displace *I. graellsii* or if it is helping *I. elegans* in the adaptation to the Iberian Peninsula conditions [26]. 

Two conclusions can be drawn from our work. First, we predict a general decrease in suitable distribution area (which would cause a shift towards northern latitudes), according to four Global Circulation Models future projections. These predicted range changes will give rise to new overlapping ranges and interspecific interactions and hybridisation between sister species. Based on previous evidence that suggests local extinction of native species in this genus [23,26,28], we predict similar extinction outcomes. Second, not only our methodology can predict hybridisation in these animals but the same principle can be applied to other taxa as long as enough information is available with regards to reproductive biology and isolation barriers.

## Supporting Information

Figure S1 **Predicted current binary (presence/absence) distribution (**A**) and predicted distribution for three time periods [2020 (**B**), 2050 (**C**), and 2080 (**D**)] under IPCC scenario A2a for *I. elegans*.** Panes B, C and D indicate for each pixel the number of binary models predicting the species as present according to four General Circulation Models (GCM), from green (1), yellow (2), orange (3) to red (4). Areas in the four shades of grey similarly represent areas that have, for one (light grey) to four (black) of the GCMs, one or more environmental variables outside the range present in the training data, and where predictions should be treated with caution.
(TIF)

Figure S2 **Predicted current binary (presence/absence) distribution (**A**) and predicted distribution for three time periods [2020 (**B**), 2050 (**C**), and 2080 (**D**)] under IPCC scenario A2a for *I. fountaineae*.** Panes B, C and D indicate for each pixel the number of binary models predicting the species as present according to four General Circulation Models (GCM), from green (1), yellow (2), orange (3) to red (4). Areas in the four shades of grey similarly represent areas that have, for one (light grey) to four (black) of the GCMs, one or more environmental variables outside the range present in the training data, and where predictions should be treated with caution.
(TIF)

Figure S3 **Predicted current binary (presence/absence) distribution (**A**) and predicted distribution for three time periods [2020 (**B**), 2050 (**C**), and 2080 (**D**)] under IPCC scenario A2a for *I. genei*.** Panes B, C and D indicate for each pixel the number of binary models predicting the species as present according to four General Circulation Models (GCM), from green (1), yellow (2), orange (3) to red (4). Areas in the four shades of grey similarly represent areas that have, for one (light grey) to four (black) of the GCMs, one or more environmental variables outside the range present in the training data, and where predictions should be treated with caution.
(TIF)

Figure S4 **Predicted current binary (presence/absence) distribution (**A**) and predicted distribution for three time periods [2020 (**B**), 2050 (**C**), and 2080 (**D**)] under IPCC scenario A2a for *I. graellsii*.** Panes B, C and D indicate for each pixel the number of binary models predicting the species as present according to four General Circulation Models (GCM), from green (1), yellow (2), orange (3) to red (4). Areas in the four shades of grey similarly represent areas that have, for one (light grey) to four (black) of the GCMs, one or more environmental variables outside the range present in the training data, and where predictions should be treated with caution.
(TIF)

Figure S5 **Predicted current binary (presence/absence) distribution (**A**) and predicted distribution for three time periods [2020 (**B**), 2050 (**C**), and 2080 (**D**)] under IPCC scenario A2a for *I. pumilio*.** Panes B, C and D indicate for each pixel the number of binary models predicting the species as present according to four General Circulation Models (GCM), from green (1), yellow (2), orange (3) to red (4). Areas in the four shades of grey similarly represent areas that have, for one (light grey) to four (black) of the GCMs, one or more environmental variables outside the range present in the training data, and where predictions should be treated with caution.
(TIF)

Figure S6 **Predicted current binary (presence/absence) distribution (**A**) and predicted distribution for three time periods [2020 (**B**), 2050 (**C**), and 2080 (**D**)] under IPCC scenario A2a for *I. saharensis*.** Panes B, C and D indicate for each pixel the number of binary models predicting the species as present according to four General Circulation Models (GCM), from green (1), yellow (2), orange (3) to red (4). Areas in the four shades of grey similarly represent areas that have, for one (light grey) to four (black) of the GCMs, one or more environmental variables outside the range present in the training data, and where predictions should be treated with caution.
(TIF)

Figure S7 **Predicted current binary (presence/absence) distribution (**A**) and predicted distribution for three time periods [2020 (**B**), 2050 (**C**) and 2080 (**D**)] under IPCC scenario A2a for *I. senegalensis*.** Panes B, C and D indicate for each pixel the number of binary models predicting the species as present according to four General Circulation Models (GCM), from green (1), yellow (2), orange (3) to red (4). Areas in the four shades of grey similarly represent areas that have, for one (light grey) to four (black) of the GCMs, one or more environmental variables outside the range present in the training data, and where predictions should be treated with caution.
(TIF)
