# Supplementary figures and images for: Climate-Induced Range Shifts and Possible Hybridisation Consequences in Insects
Source: PLoS One. 2013 Nov 15;8(11):e80531. doi: 10.1371/journal.pone.0080531 (PMC3829986; doi:10.1371/journal.pone.0080531)

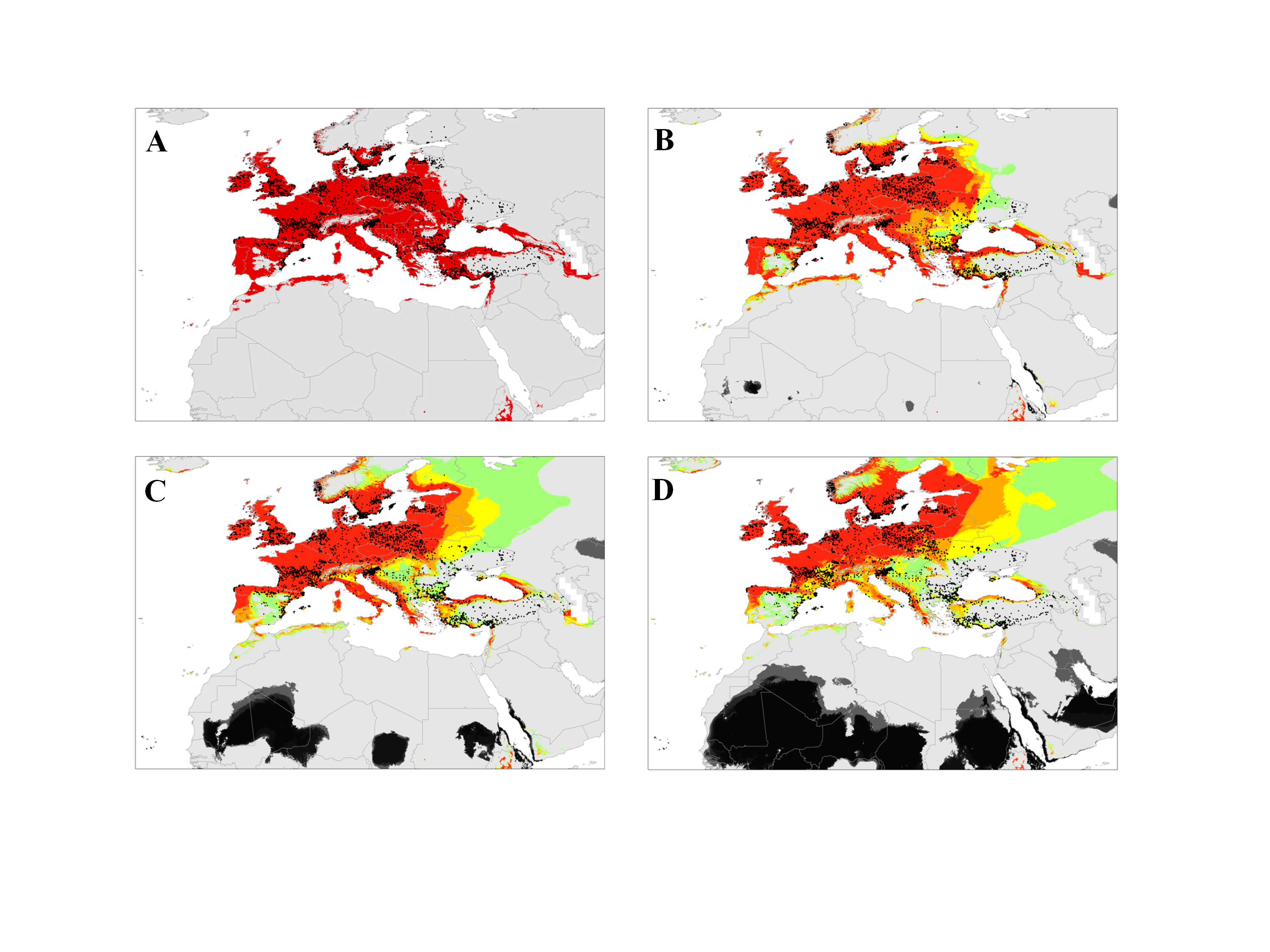

Supplement: Figure S1 — Predicted current binary (presence/absence) distribution (A) and predicted distribution for three time periods [2020 (B), 2050 (C), and 2080 (D)] under IPCC scenario A2a for I. elegans. Panes B, C and D indicate for each pixel the number of binary models predicting the species as present according to four General Circulation Models (GCM), from green (1), yellow (2), orange (3) to red (4). Areas in the four shades of grey similarly represent areas that have, for one (light grey) to four (black) of the GCMs, one or more environmental variables outside the range present in the training data, and where predictions should be treated with caution. (TIF) [file pone.0080531.s001.tif]

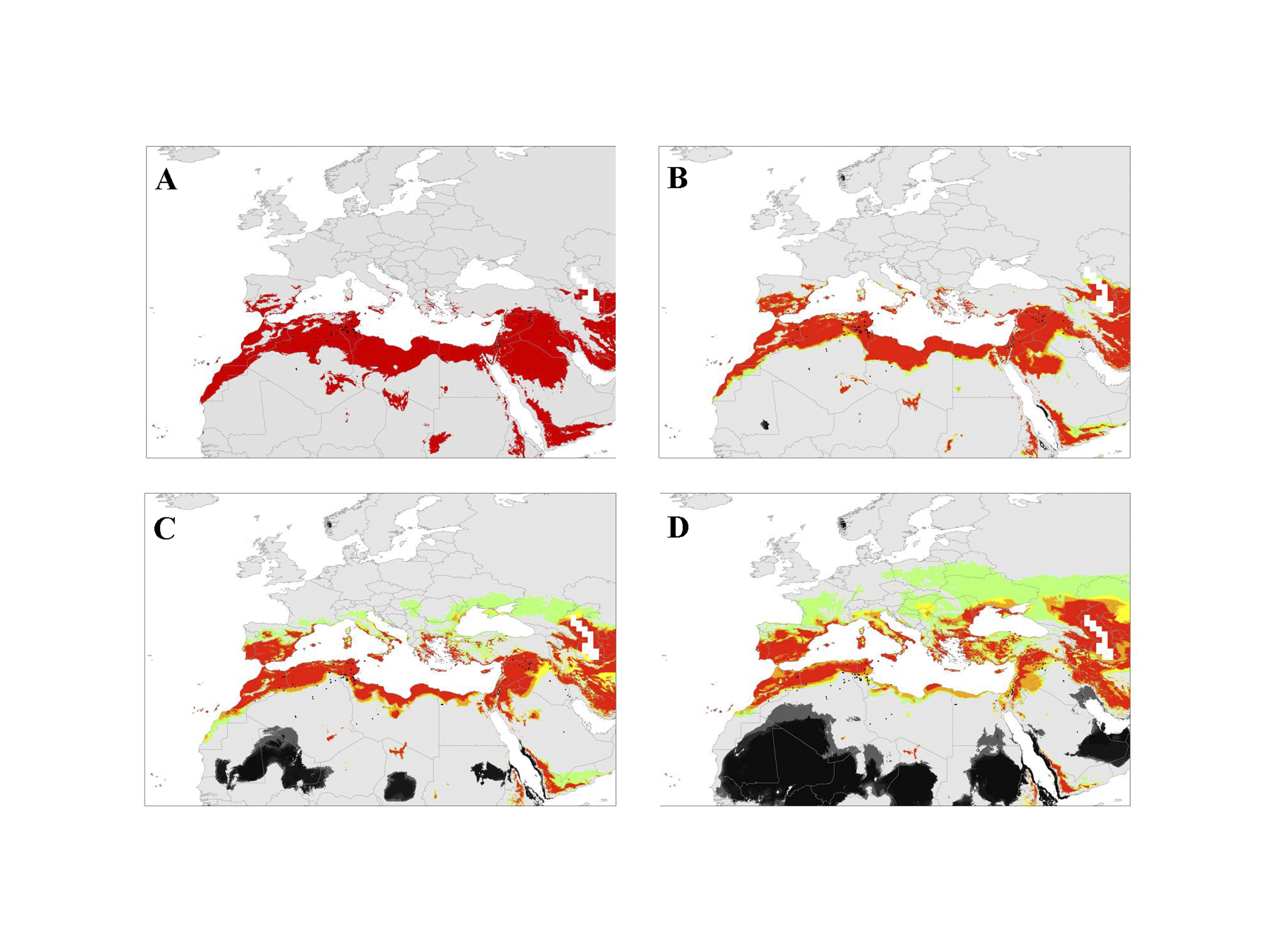

Supplement: Figure S2 — Predicted current binary (presence/absence) distribution (A) and predicted distribution for three time periods [2020 (B), 2050 (C), and 2080 (D)] under IPCC scenario A2a for I. fountaineae. Panes B, C and D indicate for each pixel the number of binary models predicting the species as present according to four General Circulation Models (GCM), from green (1), yellow (2), orange (3) to red (4). Areas in the four shades of grey similarly represent areas that have, for one (light grey) to four (black) of the GCMs, one or more environmental variables outside the range present in the training data, and where predictions should be treated with caution. (TIF) [file pone.0080531.s002.tif]

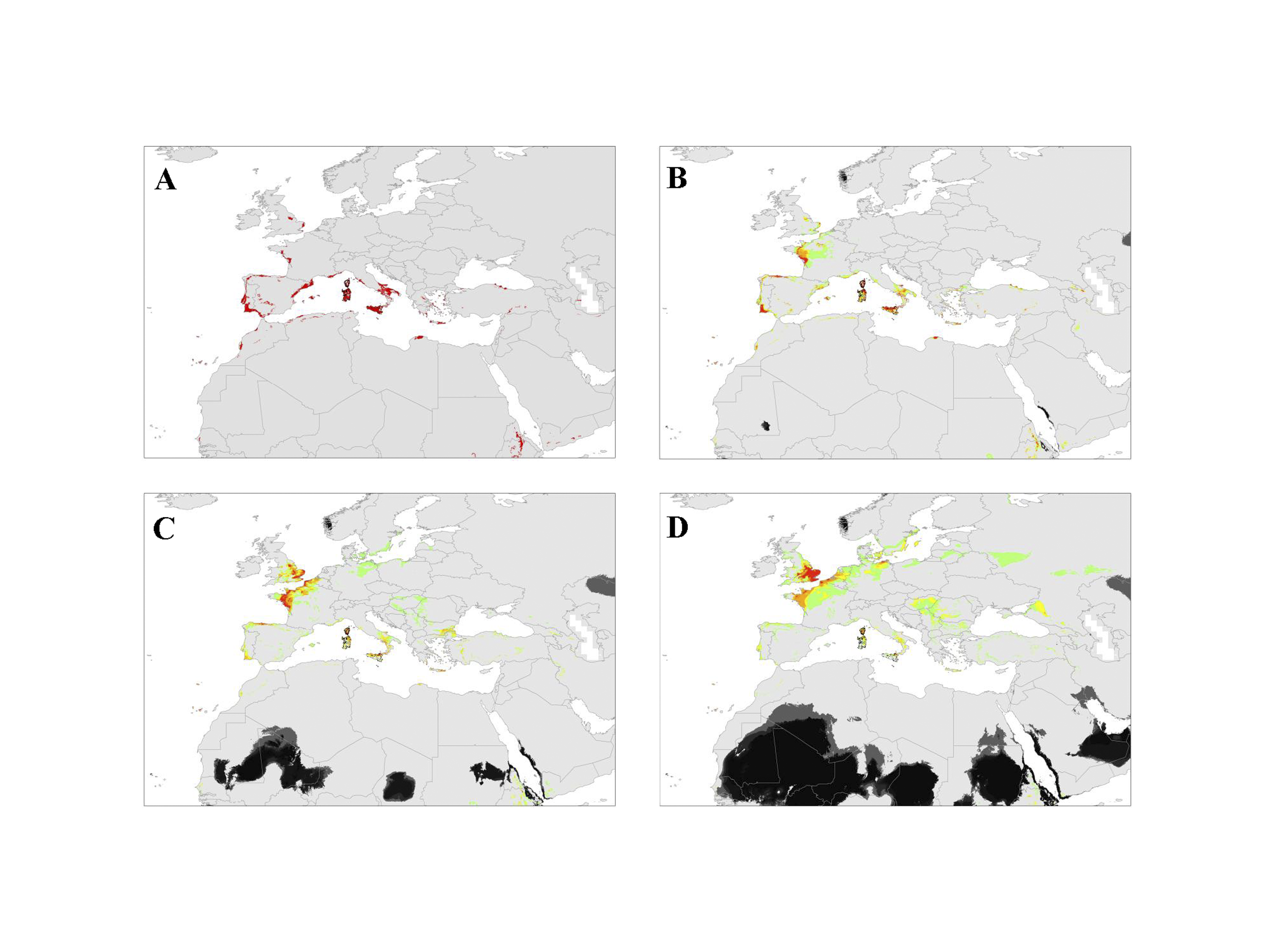

Supplement: Figure S3 — Predicted current binary (presence/absence) distribution (A) and predicted distribution for three time periods [2020 (B), 2050 (C), and 2080 (D)] under IPCC scenario A2a for I. genei. Panes B, C and D indicate for each pixel the number of binary models predicting the species as present according to four General Circulation Models (GCM), from green (1), yellow (2), orange (3) to red (4). Areas in the four shades of grey similarly represent areas that have, for one (light grey) to four (black) of the GCMs, one or more environmental variables outside the range present in the training data, and where predictions should be treated with caution. (TIF) [file pone.0080531.s003.tif]

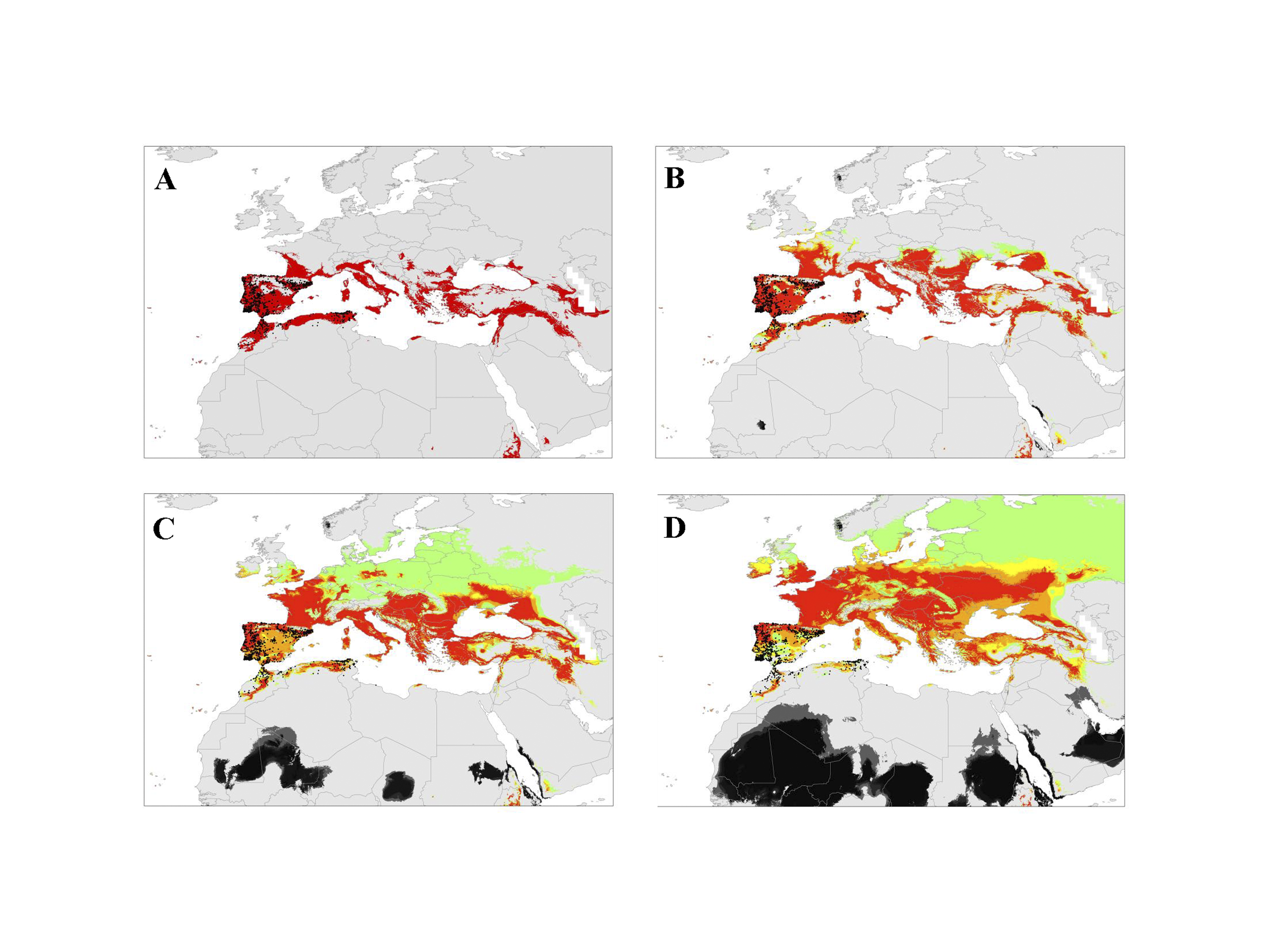

Supplement: Figure S4 — Predicted current binary (presence/absence) distribution (A) and predicted distribution for three time periods [2020 (B), 2050 (C), and 2080 (D)] under IPCC scenario A2a for I. graellsii. Panes B, C and D indicate for each pixel the number of binary models predicting the species as present according to four General Circulation Models (GCM), from green (1), yellow (2), orange (3) to red (4). Areas in the four shades of grey similarly represent areas that have, for one (light grey) to four (black) of the GCMs, one or more environmental variables outside the range present in the training data, and where predictions should be treated with caution. (TIF) [file pone.0080531.s004.tif]

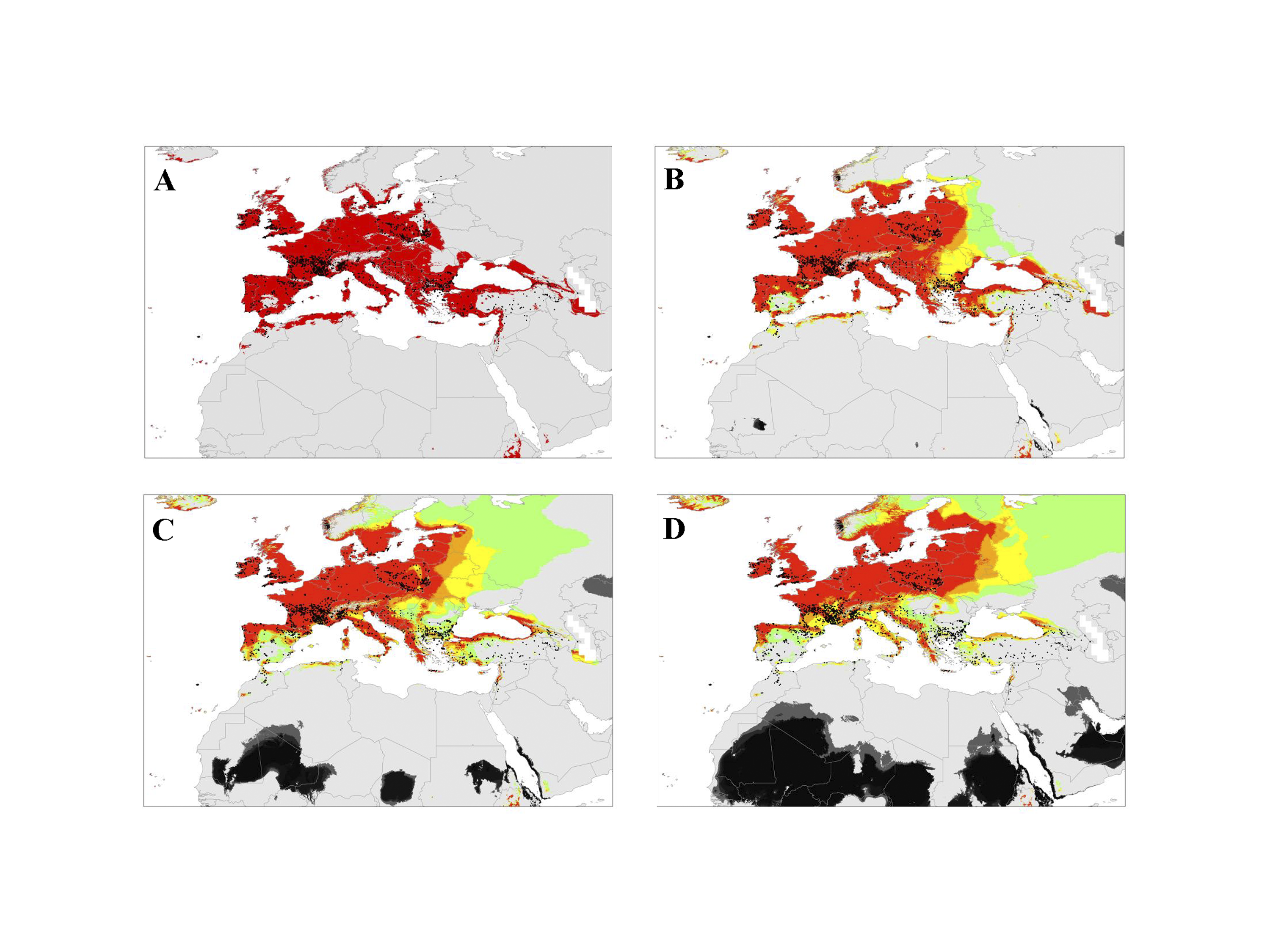

Supplement: Figure S5 — Predicted current binary (presence/absence) distribution (A) and predicted distribution for three time periods [2020 (B), 2050 (C), and 2080 (D)] under IPCC scenario A2a for I. pumilio. Panes B, C and D indicate for each pixel the number of binary models predicting the species as present according to four General Circulation Models (GCM), from green (1), yellow (2), orange (3) to red (4). Areas in the four shades of grey similarly represent areas that have, for one (light grey) to four (black) of the GCMs, one or more environmental variables outside the range present in the training data, and where predictions should be treated with caution. (TIF) [file pone.0080531.s005.tif]

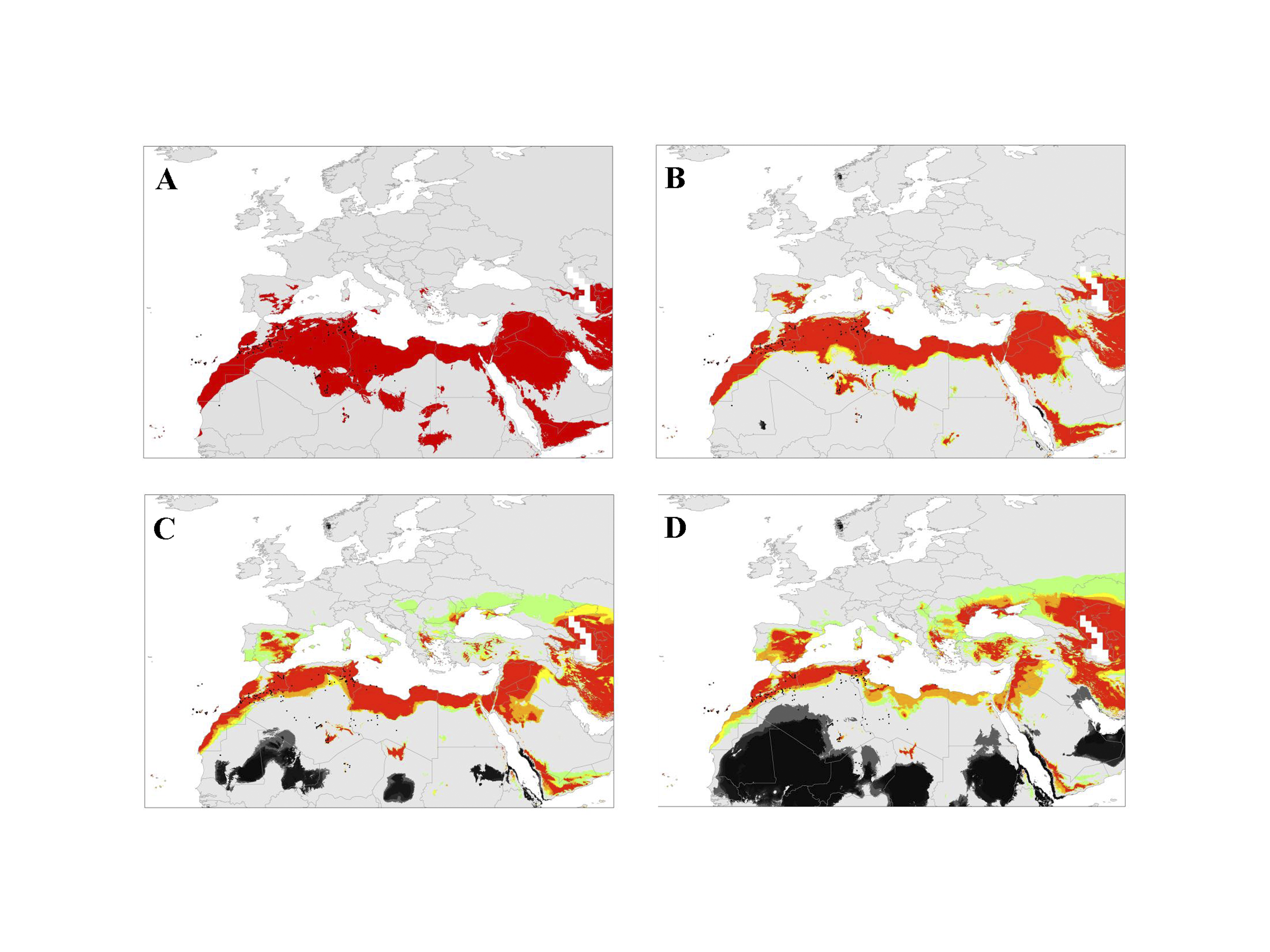

Supplement: Figure S6 — Predicted current binary (presence/absence) distribution (A) and predicted distribution for three time periods [2020 (B), 2050 (C), and 2080 (D)] under IPCC scenario A2a for I. saharensis. Panes B, C and D indicate for each pixel the number of binary models predicting the species as present according to four General Circulation Models (GCM), from green (1), yellow (2), orange (3) to red (4). Areas in the four shades of grey similarly represent areas that have, for one (light grey) to four (black) of the GCMs, one or more environmental variables outside the range present in the training data, and where predictions should be treated with caution. (TIF) [file pone.0080531.s006.tif]

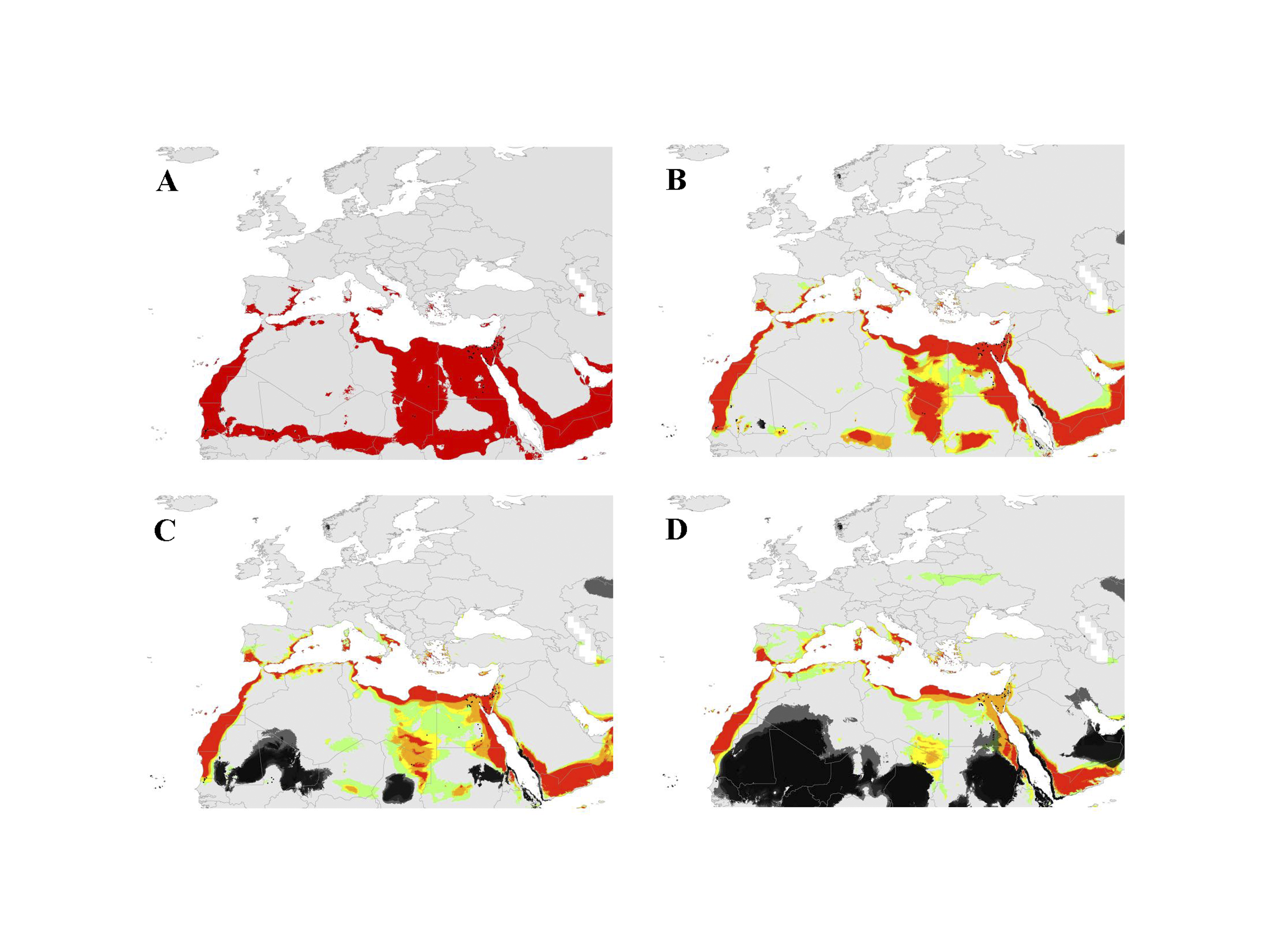

Supplement: Figure S7 — Predicted current binary (presence/absence) distribution (A) and predicted distribution for three time periods [2020 (B), 2050 (C) and 2080 (D)] under IPCC scenario A2a for I. senegalensis. Panes B, C and D indicate for each pixel the number of binary models predicting the species as present according to four General Circulation Models (GCM), from green (1), yellow (2), orange (3) to red (4). Areas in the four shades of grey similarly represent areas that have, for one (light grey) to four (black) of the GCMs, one or more environmental variables outside the range present in the training data, and where predictions should be treated with caution. (TIF) [file pone.0080531.s007.tif]
